# Synergic action of L-acetylcarnitine and L-methylfolate in Mouse Models of Stress-Related Disorders and Human iPSC-Derived Dopaminergic Neurons

**DOI:** 10.3389/fphar.2022.913210

**Published:** 2022-06-02

**Authors:** Rosamaria Orlando, Roxana Paula Ginerete, Laura Cavalleri, Vincenza Aliperti, Tiziana Imbriglio, Giuseppe Battaglia, Anna Rita Zuena, Ferdinando Nicoletti, Emilio Merlo Pich, Ginetta Collo

**Affiliations:** ^1^ IRCCS Neuromed, Pozzilli, Italy; ^2^ Department of Physiology and Pharmacology, Sapienza University, Rome, Italy; ^3^ Department of Molecular and Translational Medicine, University of Brescia, Brescia, Italy; ^4^ Research and Development, Alfasigma S.p.A., Bologna, Italy

**Keywords:** depression, chronic stress, hiPSC, L-acetylcarnitine, L-methylfolate, BDNF, mGlu2 receptors

## Abstract

The epigenetic agents, L-acetylcarnitine (LAC) and L-methylfolate (MF) are putative candidates as add-on drugs in depression. We evaluated the effect of a combined treatment with LAC and MF in two different paradigms of chronic stress in mice and in human inducible pluripotent stem cells (hiPSCs) differentiated into dopaminergic neurons. Two groups of mice were exposed to chronic unpredictable stress (CUS) for 28 days or chronic restraint stress (CRS) for 21 day, and LAC (30 or 100 mg/kg) and/or MF (0.75 or 3 mg/kg) were administered i.p. once a day for 14 days, starting from the last week of stress. In both stress paradigms, LAC and MF acted synergistically in reducing the immobility time in the forced swim test and enhancing BDNF protein levels in the frontal cortex and hippocampus. In addition, LAC and MF acted synergistically in enhancing type-2 metabotropic glutamate receptor (mGlu2) protein levels in the hippocampus of mice exposed to CRS. Interestingly, CRS mice treated with MF showed an up-regulation of NFκB p65, which is a substrate for LAC-induced acetylation. We could also demonstrate a synergism between LAC and MF in cultured hiPSCs differentiated into dopamine neurons, by measuring dendrite length and number, and area of the cell soma after 3 days of drug exposure. These findings support the combined use of LAC and MF in the treatment of MDD and other stress-related disorders.

## Introduction

Nearly 10–30 percent of patients affected by major depressive disorder (MDD) do not respond adequately to conventional antidepressant drugs (e.g., selective serotonin reuptake inhibitors or serotonin/noradrenalin reuptake inhibitors), showing either a partial response or no response at all. Such suboptimal response is called treatment-resistant depression (TRD), conventionally defined as an insufficient response to at least two different antidepressants administered at adequate dose and duration (Heerlein et al., 2021). Several factors might contribute to non-response and low remission rates of MDD, including poor long-term adherence to pharmacological therapies and discontinuation due to adverse events. The NMDA receptor antagonist (S)-ketamine, is a new option for TRD, but concerns remain about the safety and tolerability profile of the drug (reviewed by McIntyre et al., 2021). Thus, there is an urgent need for innovative treatment strategies to achieve full functional recovery in patients affected by MDD, also considering the impact of COVID-19 pandemic on mental health (COVID-19 Mental Disorders Collaborators, 2021).

Epigenetic modifications, mainly changes in DNA methylation and histone acetylation, have been consistently associated with depression in animal models (rodent and primates) and humans (reviewed by Nestler et al., 2016; Saavedra et al., 2016; Han and Nestler, 2017; Torres-Berrío et al., 2019). Hence, targeting epigenetic mechanisms has been considered as a new valuable approach in the treatment of MDD and TRD. The epigenetic agents, L-acetylcarnitine (LAC) and L-methylfolate (MF) are putative candidates as add-on drugs in depression.

LAC is a small endogenous molecule of growing interest for its biological and pharmacological effects. LAC is widely distributed in many tissues, including brain, and the carnitine moiety of the molecule supports cell energy metabolism acting as a transporter of fatty acids across the mitochondrial membranes (reviewed by Chiechio et al., 2017). LAC has a clinical indication in the treatment of neuropathic pain (Watson and Dyck, 2015). Preclinical studies have consistently shown that LAC-induced analgesia is mediated by the epigenetic up-regulation of type-2 metabotropic glutamate receptors (mGlu2 receptors), which results from LAC-induced acetylation of the p65NFκB transcription factor and H3 histone acetylation (Chiechio et al., 2002; Chiechio et al., 2006; Notartomaso et al., 2017). These findings laid the groundwork for the study of LAC in rat and mouse models of depression. LAC treatment was shown to induce a rapid and sustained antidepressant-like activity in spontaneously depressed Flinders Sensitive Line (FSL) rats and in mice exposed to chronic unpredictable stress (Cuccurazzu et al., 2013; Nasca et al., 2013), as a result of the epigenetic up-regulation of mGlu2 receptors and brain-derived neurotrophic factor (BDNF). In more recent studies, LAC treatment was consistently shown to enhance resilience to stress and display antidepressant-like activity in mice (Bigio et al., 2016; Lau et al., 2017; Nasca et al., 2017; Cherix et al., 2020). Interestingly, endogenous LAC levels were reduced in patients affected by MDD, and the extent of reduction was associated with the history of childhood trauma in patients with TRD (Nasca et al., 2018). LAC treatment showed efficacy in patients affected by persistent depressive disorder (previously called “dysthymia”) (Zanardi and Smeraldi, 2006; Bersani et al., 2013), and, in one study, the drug showed a shorter clinical latency and better profile of tolerability compared to fluoxetine (Bersani et al., 2013).

Folate deficiency has been implicated in the pathophysiology of depression (Lazarou and Kapsou, 2010; Bender et al., 2017). In addition, a variety of controlled and open-label studies have shown efficacy of folic acid and its metabolic product, MF, in improving depressive symptoms, when administered as add-on agents in patients with normo- and hypofolatemic status (reviewed in Altaf et al., 2021). A retrospective analysis comparing patients under treatment with SSRI or SNRI antidepressants alone or in combination with MF, found a greater efficacy of the combination in improving depressive symptoms and reducing drop-out from medication (Papakostas et al., 2014; reviewed by; Jain et al., 2020). MF can cross the blood–brain barrier, and is transformed by the enzyme methylentetrahydrofolate reductase (MTHFR) into the methylating agent, 5,10-methylentetrahydrofolate, or, alternatively is converted into tetrahydrofolate. These metabolic pathways are associated with the conversion of dihydrobiopterine (BH2) into tetrahydrobiopterine (BH4), and the conversion of homocysteine into methionine, respectively. BH4 is a cofactor of both tyrosine hydroxylase and tryptophan hydroxylase, which are key enzymes in catecholamine and serotonin synthesis, respectively, while methionine is the direct precursor of S-adenosylmethionine (SAM), which acts as a methyl donor (Leung et al., 2013; Lam et al., 2022). Thus, MF may regulate gene expression by affecting the methylation status of DNA and histones (Leung et al., 2013). There are no studies with MF in preclinical models of depression or other stress-related disorders. However, treatment with its precursor, folic acid, was shown to reduce depressive-like behaviour caused by chronic administration of corticosterone (Rosa et al., 2014) or acute restraint stress (Budni et al., 2013) in mice. In addition, folic acid treatment was shown to improve depressive- and anxiety-like behaviours and reduce the number of activated microglia and astrocytes in a mouse model of postnatal immune activation (Zhao et al., 2022). Folic acid could also up-regulate BDNF expression in cultured astrocytes through an epigenetic mechanism mediated by trimethylation of histone H3 lysine 27 (Zhao et al., 2022).

Here, we examined the combined effect of LAC and MF on depressive-like behaviour to establish whether the convergence of two epigenetic mechanisms might be therapeutically valuable. We extended the analysis to BDNF and mGlu2 receptor expression in the frontal cortex and hippocampus, which are key regions in the pathophysiology of depression. An impairment of BDNF signaling is involved in the pathophysiology of depression, and mechanisms of BDNF-dependent neuronal plasticity have been consistently implicated in the response to antidepressant medication (reviewed by Castrén and Monteggia, 2021). mGlu2 receptors are induced by LAC, and may shape the response to stress (see above). In addition, we examined the effect of LAC and MF on human dopaminergic neurons, which are affected in mood disorders, using a translational strategy based on human inducible pluripotent stem cell (hiPSC) (Cavalleri et al., 2018).

## Materials and Methods

### Drugs and Treatments

L-acetyl-carnitine (LAC) and MF (kindly provided by Alfasigma S.p.A, Bologna, Italy), were dissolved in saline and injected i.p. once a day, for 14 days.

### Animals and Drugs

Seven weeks old male C57BL/6J mice were obtained from Charles River (Calco, Italy) and were housed under controlled conditions (temperature 22 ± 1°C and humidity 40%) with a 12 h light/dark cycle and food and water *ad libitum*. Mice were left undisturbed for 1 week before beginning any experimental procedure. All experiments were performed according to the European (86/609/EEC) and Italian (D. Lgs. 26/2014) guidelines of animal care and approved by the Neuromed Institutional Animal Care and Use Committee and by the Italian Ministry of Health (protocol # 1110/2020-PR). Any effort was made to minimize animal suffering and to reduce the number of animals used.

### Experimental Design

We tested the antidepressant-like activity of LAC and MF using two established models of depression in mice: 1) exposure to chronic unpredictable stress (CUS); and 2) exposure to chronic restraint stress (CRS). Eight weeks old male mice were used in both models. All mice were randomly allocated to CUS/CRS or control (Ctrl) groups.

Mice in the CUS or CRS groups were housed in standard mouse cages (45 cm × 28 cm × 13 cm), four mice per cage. Mice in the Ctrl group were housed in large cages (59.5 cm × 38 cm × 20 cm) with environmental enrichment (small plastic and cardboard houses, cardboard tube, paper sizzle nest material) in a separate room from the stress group area.

### Chronic Stress Procedure

In CUS group, mice were subjected to various unpredictable stressors for 28 consecutive days. We followed the previously published CUS protocol (di Nuzzo et al., 2015). The stress protocol consisted of 1–3 h sessions in the morning and an overnight session. The following stressors were included in the protocol: food deprivation for 12 h; 45° cage tilt for 12 h; wet bedding (250 ml of water in 750 ml of bedding) for 12 h; overnight illumination; 1-h restraint stress in a 15 × 5-cm cylinder; cage rotation for 1 h; different partner for 3 h; stroboscopic light exposure overnight; overcrowding for 12 h; and light off for 3 h. CUS animals were randomly exposed to any two stressors (day and night stressor) daily for 28 days. Mice exposed to CUS were chronically treated i.p. with saline, LAC 30 or 100 mg/kg, MF 0.75 or 3 mg/kg, or the combination of LAC 100 mg/kg + MF 0.75 mg/kg, LAC 100 mg/kg + MF 3 mg/kg, or LAC 30 mg/kg + MF 3 mg/kg for 9 or 14 days, starting from the last week of CUS.

In the CRS paradigm, mice were placed in the restrainers for 2 h per day in the morning for 21 consecutive days. The restraint device contained two 0.4-cm air holes and allowed mice to stretch their legs but did not permit locomotion within the tube (Lau et al., 2017). Naïve age-matched not-stressed animals were used as controls. Ctrl mice were maintained undisturbed in their home cages. CRS mice were chronically treated i.p. for 14 days, starting on day 14 of CRS, with saline, LAC (30 mg/kg), MF (3 mg/kg) or the combination of LAC (30 mg/kg) and MF (3 mg/kg).

### Behavioural Test

#### Forced Swim Test

We used the forced swim test (Porsolt et al., 2001) for the assessment of depressive-like behaviour. The test was performed in the morning (from 9:30 to 12a.m.). Mice were allowed to adapt to the experimental room 60 min before testing and were then placed individually into glass cylinders (22 cm diameter, 24 cm high) filled with 15 cm deep water at 25–27°C, 30 min after being treated. Mice were videotaped during a 6 min session. The immobility time (in sec) during the last 4 min of the test was scored by a blind observer. Mice were considered “immobile” if they showed only minimal movements to keep the head above water or floating (di Nuzzo et al., 2015). All mice were killed 24 h after the last FST session for biochemical analysis.

### Western Blot Analysis

Mice were killed by rapid decapitation, and hippocampi and frontal cortex were dissected, flash frozen, and stored at −80°C until processing. Tissues were homogenized at 4°C in ice-cold lysis buffer, and 2 μL of homogenates were used for protein determinations. Proteins (15–25 μg) were resuspended in sodium dodecyl sulphate (SDS)-bromophenol blue reducing buffer containing 5% 2-mercaptoethanol and separated by electrophoresis on 8% (for mGlu2 receptor) or 12% (for BDNF) SDS-polyacrylamide gels. Samples were not boiled (for mGlu receptors), or were incubated at 65°C for 5 min (for BDNF), before loading. Gels were electroblotted on PVDF membranes and filters were blocked in 5% non-fat dry milk solution (60 min at room temperature). Blots were incubated overnight at 4°C with rabbit polyclonal anti-BDNF antibodies (Invitrogen, code: PA5-85730; 1:1000), rabbit polyclonal anti-mGlu2 receptor antibodies (Abcam, code: ab150387; 1:10,000), rabbit polyclonal anti-p65/NFκB antibodies (Santa Cruz Biotechnology; code: sc-372; 1:500) and mouse monoclonal anti-GAPDH antibodies (Santa Cruz Biotechnology, code: sc-32233; 1:1000). Membranes were rinsed for 5 min, 4 times, at RT in TBS-T, and then incubated with secondary peroxidase-coupled antibodies (1:7000, Millipore). Immunostaining was revealed by enhanced chemiluminescence luminosity.

### Dopaminergic Differentiation of Human iPSCs

Human iPSCs (hiPSCs) (Collo et al., 2018) were induced to differentiate into midbrain dopamine (DA) neurons using the dual SMAD inhibition protocol described by Kriks and others (Kriks et al., 2011) with some modifications (Fedele et al., 2017). In brief, hiPSCs were cultured on Matrigel-coated (BD Biosciences) plates in mTeSR1 medium (StemCell Technologies). For midbrain DA neuron induction, hiPSCs were dissociated with Accutase™ (StemCell Technologies) and seeded (3 × 10^4^ cells/cm2) on Matrigel-coated plates in Knockout Serum Replacement (KSR) medium containing Knockout™ DMEM, 15% KSR, GlutaMAX™ and 10 μm 2-mercaptoethanol, in the presence of LDN193189 (0.1 µm, Stemgent), SB431542 (10 μm, Tocris Bioscience), Shh C25II (0.1 μg/ml, R&D Systems), Purmorphamine (2 μm, Stemgent), Fibroblast Growth Factor 8 (0.1 μg/ml, R&D Systems), and CHIR99021 (3μm, Stemgent). From day 5, the KSR medium was gradually shifted to the N2 medium (Knockout™ DMEM/F12, N2 supplement and GlutaMAX™, all from Gibco-Invitrogen). On day 11, the medium was changed into Neurobasal/B27/GlutaMAX™ supplemented with CHIR99021, brain-derived neurotrophic factor (BDNF, 20 ng/ml, R&D Systems), ascorbic acid (AA, 0.2 mm, Sigma-Aldrich), dibutyryl cAMP (cAMP, 0.5 mm, Sigma-Aldrich), transforming growth factor type β3 (TGFβ3, 1 ng/ml, R&D Systems), glial cell line-derived neurotrophic factor (GDNF, 20 ng/ml, R&D Systems) and DAPT (10 nm, Tocris Bioscience). On day 21, cells were seeded on plates pre-coated with Polyornithine (15 μg/ml, Sigma-Aldrich), Fibronectin (2 μg/ml, Sigma-Aldrich) and Laminin (1 μg/ml, Sigma-Aldrich), and co-cultured with mouse primary cortical astrocytes. Human iPSC-derived DA neurons were used for pharmacological studies, immunocytochemistry and morphological analysis starting from day 40.

### *In vitro* Pharmacological Treatments

LAC and MF were diluted in water. For each vehicle treatment, the solvent required by the specific drug was used at the same dilution used for the active treatment. Pharmacological treatments were performed from day 40 in culture. Three days before performing the pharmacological treatment, grow factors and small molecules (BDNF, AA, cAMP, TGFβ3, GDNF, and DAPT) were gradually removed. Neuronal cultures were incubated with LAC at concentrations of 1, 10, or 50 μm and with MF at the concentrations of 0.1, 1, or 5 μm for 72 h. In parallel samples neuronal cultures were exposed to vehicle. Neurons were maintained in culture until fixation, which was performed at 72 h.

### Immunocytochemistry

Immunocytochemistry of DA neurons for morphological analysis was performed using anti-tyrosine hydroxylase (TH) rabbit polyclonal antibody (Millipore; catalog # AB152; dilution 1:800), followed by incubation with a biotinylated goat anti-rabbit antibody (Amersham; catalog # RPN1004; dilution 1:500), as previously described (Collo et al., 2008; Collo et al., 2013).

### Computer-Assisted Morphological Analysis

Digital images were acquired with an Olympus IX51 microscope connected to an Olympus (Hamburg, Germany) digital camera and a PC. Morphometric measurements were performed by a blinded examiner on digitalized images using Image-Pro Plus software (Media Cybernetics, Bethesda, MD). Morphological indicators of structural plasticity were considered: 1) the maximal dendrite length, 2) the number of primary dendrites and 3) the soma area (Collo et al., 2008). Maximal dendrite length was defined as the distance from the soma (hillock base) to the tip of the longest dendrite for each neuron; dendrites shorter than 20 µm were excluded from the analysis. Primary dendrites were defined as those directly stemming from the soma. Soma area was assessed by measuring the surface (µm^2^) included by the external perimeter drawn on the cell membrane of neurons identified by TH^+^ staining. Two cover slides per treatment groups were examined so to obtain measurements from at least 70–100 neurons.

### Statistical Analysis

For *in vivo* studies, data were expressed as means ± S.E.M. Statistical analysis was performed with GraphPad Prism software (Version 7; San Diego, CA, United States). Student’s t-test was used for comparisons between 2 groups (Figure 4A), and one-way analysis of variance (ANOVA) + Fisher’s LSD was employed to compare more sets of data. A statistical significance was set at *p* < 0.05.

For the *in vitro* studies, data were expressed as mean ± S.E.M. Significant differences from control conditions were determined using One-way analysis of variance (ANOVA) + Fisher’s LSD provided by GraphPad Prism software.

## Results

### Synergism Between L-acetylcarnitine and L-methylfolate in Mouse Models of Stress-Related Disorders

**FIGURE 1:**
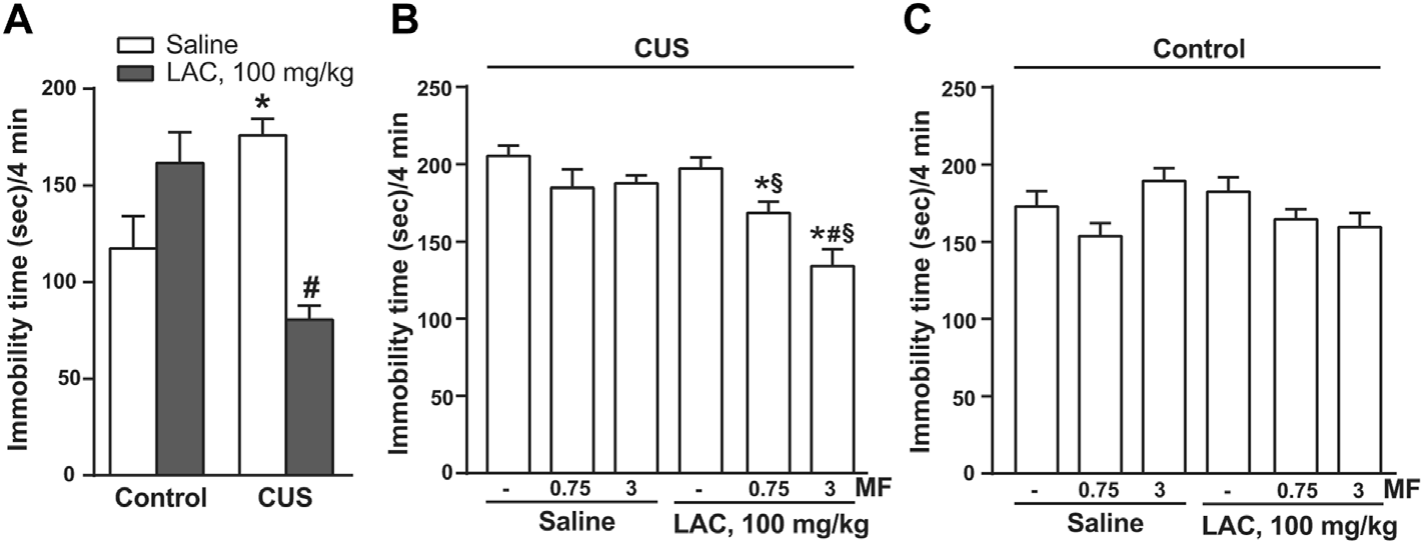
Synergism between MF and “anti-depressive doses” of LAC in reducing the immobility time in mice subjected to CUS. In **(A)**, either unstressed (Ctrl) or CUS mice were treated i.p. with LAC (100 mg/kg) or saline for 9 days. In **(B)** or **(C)**, CUS **(B)** or Ctrl **(C)** mice were treated for 3 days with either saline or LAC (100 mg/kg) combined or not with MF (0.75 or 3 mg/kg, i.p.). Data are means ± SEM. One-way ANOVA + Fisher’s LSD. **(A)** *p < 0.05 vs. Ctrl saline; #*p* < 0.05 vs. CUS saline and Ctrl LAC100 mg/kg; F_(3,26)_ = 11.09 (*n* = 7–8). **(B)** *p < 0.05 vs. saline and LAC alone; #*p* < 0.05 vs. LAC + MF 0.75, vs. MF 3 mg/kg, and vs. MF 0.75; F_(5,41)_ = 9.542 (*n* = 8). Neither LAC nor MF alone or in combination caused changes in the immobility time in unstressed mice after 3 days of treatment **(C)** (*n* = 8).

We used two paradigms of exposure to stress (CUS or CRS) to study the antidepressant-like activity of LAC and MF. In two independent experiments, mice were exposed to CUS for 28 days, and injected daily with LAC, MF, or their combination for either 9 or 14 days, starting from the last week of stress exposure. The forced swim test was used for the assessment of depressive-like behaviour after 3, 9 or 14 days of drug treatment. In the first experiment, LAC was used at the dose of 100 mg/kg, i.p, which is known to display antidepressant-like activity in rodents (Nasca et al., 2013; Nasca et al., 2018; Cherix et al., 2020). In this experiment, LAC treatment was not extended beyond 9 days because of the lockdown associated with COVID-19 pandemic. Figure 1A shows the immobility time in control and CUS mice treated i.p. for 9 days with either saline or standard doses of LAC (100 mg/kg). LAC treatment was highly effective in reducing the immobility time in the forced swim test in stressed mice, but not in unstressed controls (Figure 1A). Figures 1B,C show the immobility time of either CUS (Figure 1B) or control (Figure 1C) mice treated for 3 days with saline or LAC (100 mg/kg) combined or not with 0.75 or 3 mg/kg MF. In mice treated with saline alone, exposure to CUS significantly enhanced the immobility time (head-to-head comparison between the first two bars of Figure 1B,C: t_14_ = 2.685; *p* = 0.0178; Student’s t test). LAC treatment did not display antidepressant-like effects in stressed mice after 3 days of treatment (Figure 1B). However, at this time point, LAC became effective when combined with MF at doses of either 0.75 or 3 mg/kg, which were inactive on their own. The synergism between the two drugs was prominent using 3 mg/kg of MF, a dose that we used in all other experiments (Figure 1B). Neither LAC nor MF alone or in combination caused changes in the immobility time in unstressed mice after 3 days of treatment (Figure 1C).

**FIGURE 2:**
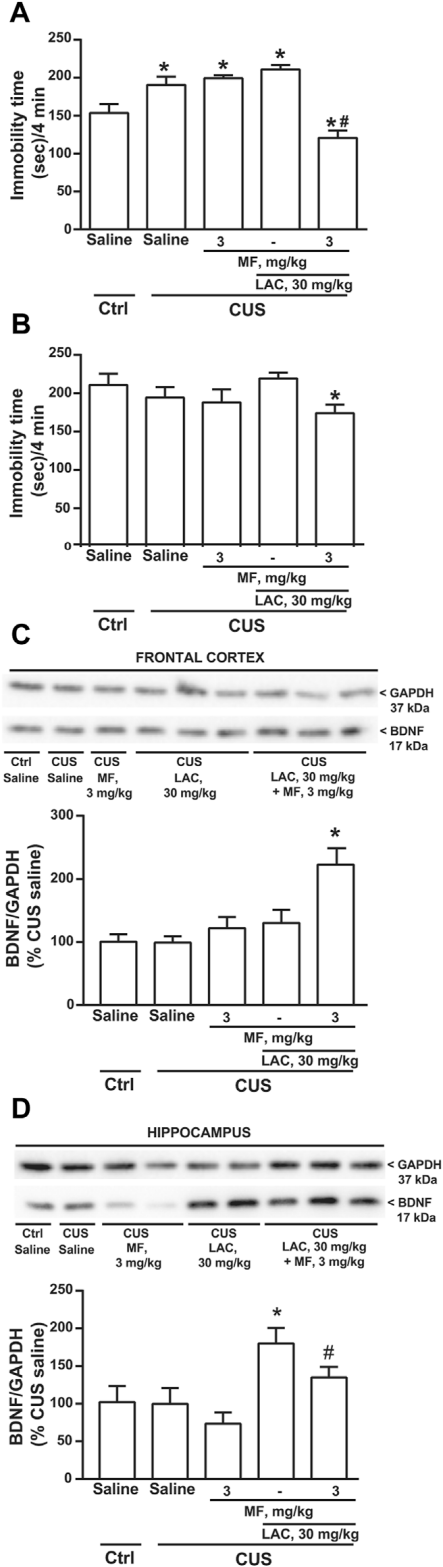
Synergism between MF and sub-threshold doses of LAC in reducing the immobility time and enhancing BDNF expression in mice subjected to CUS. Immobility time in the forced swim test in unstressed (Ctrl) mice treated i.p. for 3 days with saline and CUS mice treated i.p. for 3 days with saline, MF (3 mg/kg), LAC (30 mg/kg) or LAC + MF is show in **(A)**. BDNF protein levels in the frontal cortex ad hippocampus are shown in **(C)** and **(D)**, respectively, where immunoblots are representative of two blots used for the analysis. Data are means ± SEM. One-way ANOVA + Fisher’s LSD **(A)**, *p* < 0.05 vs. Ctrl saline (*) or vs. all other groups (#), F_*(4,35)*_ = 17.19; *p* < 0.0001 (*n* = 8) **(B)**, *p < 0.05 vs. CUS LAC, F_*(4,35)*_ = 1.828. *p* = 0.1455 (*n* = 8) **(C)**, *p < 0.05 vs. all other groups, F_*(4,20)*_ = 7.459; *p* = 0.0008 (*n* = 4–6) **(D)**, *p* < 0.05 vs. Ctrl saline, CUS saline, and CUS MF (*) or vs. CUS MF (#), F_*(4,16)*_ = 3.472; *p* = 0.0318 (*n* = 2–5).

In the second experiment, mice were exposed to CUS for 28 days, and either LAC and/or MF were injected daily for 14 days. In this experiment, LAC was used at the sub-threshold dose of 30 mg/kg, to avoid a ceiling effect of LAC at 14 days of treatment. MF was used at the dose of 3 mg/kg. Only one group of unstressed mice treated daily with saline was included in the experiment. After 3 days, CUS mice treated with saline showed a significant increase in the immobility time with respect to unstressed mice treated with saline (Figure 2A). LAC or MF alone were inactive in CUS mice, whereas their combination significantly reduced the immobility time (Figure 2A). After 14 days, there was no difference between CUS and unstressed mice treated with saline, perhaps reflecting habituation to the test or the 7 days of lag time after the end of CUS. However, the combination of 30 mg/kg of LAC with 3 mg/kg of MF significantly reduced the immobility time with respect to CUS mice treated with LAC alone (Figure 2B). All mice of this second experiments were killed at the end of drug treatment (i.e., 7 days after the end of CUS), and used for the assessment of BDNF protein levels in the frontal cortex and hippocampus. Western blot analysis showed a single band at 17 kDa, corresponding to the mature form of BDNF (Figures 2C,D). In both brain regions, no changes in BDNF protein levels were found in stressed mice treated with saline as compared to unstressed mice treated with saline (Figures 2C,D). Data obtained with drug treatments in the two regions were not uniform. In the frontal cortex, only the combined treatment of LAC (30 mg/kg, i.p.) with MF (3 mg/kg, i.p.) caused a substantial increase in BDNF levels in stressed mice (Figure 2C). In contrast, in the hippocampus, treatment with LAC alone was able to enhance BDNF levels, and the effect of LAC was not potentiated by MF (Figure 2D).

**FIGURE 3:**
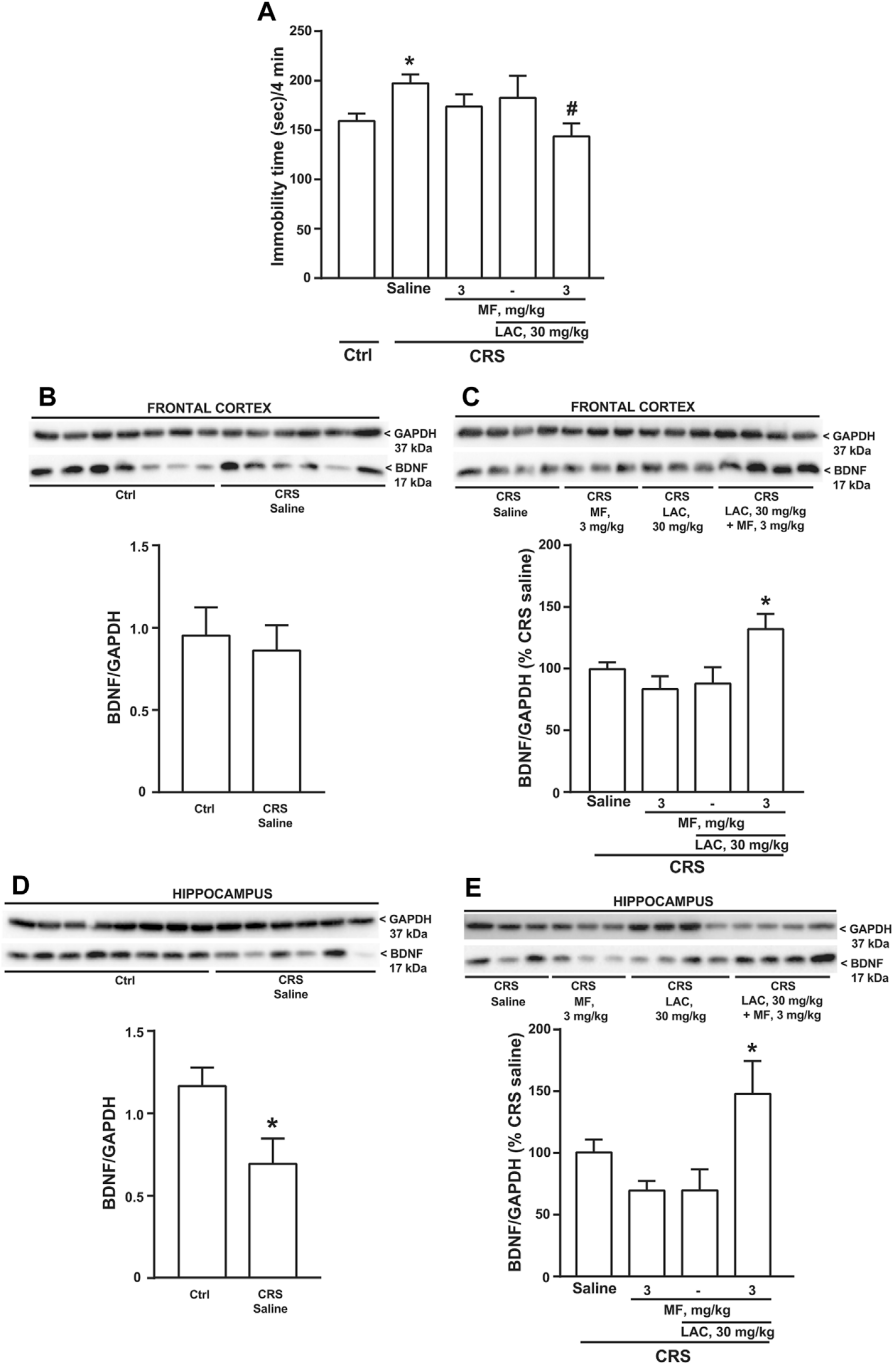
Synergism between MF and sub-threshold doses of LAC in reducing the immobility time and enhancing BDNF expression in mice subjected to CRS. Immobility time in the forced swim test in unstressed control (Ctrl) mice and in mice exposed to CRS for 21 days, after 14 days of treatment with saline, MF (3 mg/kg), LAC 30 (mg/kg) or their combination is shown in **(A)**. BDNF protein levels in the frontal cortex ad hippocampus are shown in **(B,C,D,E)**, respectively. In **(C,E)**, immunoblots are representative of two blots used for the analysis. Data are means ± SEM (*n* = 8–15 in A; 6–7 in B; and 6–8 in **(C–E)**. One-way ANOVA + Fisher’s LSD **(A)**, *p* < 0.05 vs. saline (*) or vs. CRS saline (#), F_(4,41)_ = 2.420; *p* = 0.0637 **(C)**, *p < 0.05 vs. all other groups, F_(3,24)_ = 4.504; *p* = 0.0121, and **(E)**, F_(3,24)_ = 4.171; *p* = 0.0164. Student’s t test **(D)** t_12_ = 2.551; *p* = 0.0254.

We used CRS as a second paradigm of stress. Mice were exposed to CRS for 21 days and LAC (30 mg/kg, i.p.), MF (3 mg/kg, i.p.) or their combination were injected daily for 14 days starting from the last week of stress exposure. The forced swim test performed at the end of drug treatment showed an increased immobility time in CRS mice treated with saline as compared to unstressed controls treated with saline. Neither LAC nor MF alone were active in CRS mice, whereas their combination significantly reduced the immobility time (Figure 3A), similarly to what observed in CUS mice (see above). BDNF levels were unchanged in the frontal cortex in CRS mice treated with saline as compared to control mice (Figure 3B). However, as opposed to data obtained in the CUS model, BDNF levels were reduced in the hippocampus of CRS mice (Figure 3D). In both regions, BDNF levels were unaffected by LAC or MF alone, but were largely increased by the combination of the two drugs (Figures 3C,E).

**FIGURE 4:**
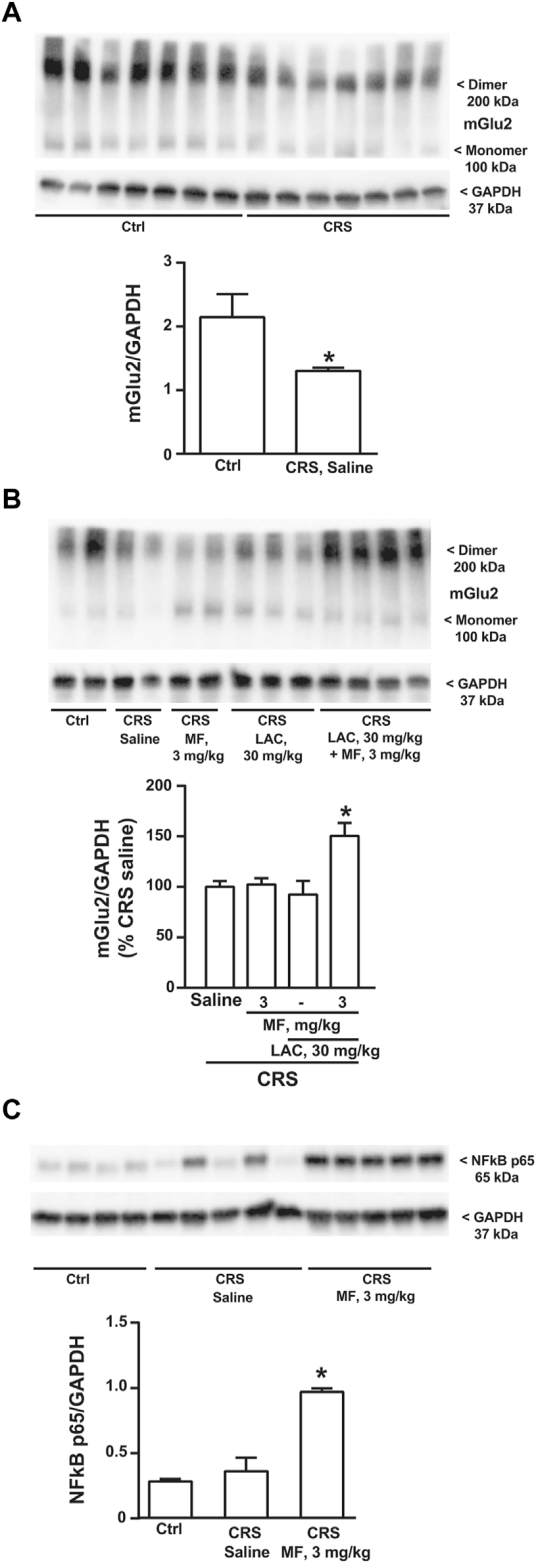
Immunoblot analysis of mGlu2 receptor and NFκB p65 protein levels in the hippocampus. mGlu2 protein levels in the hippocampus of CRS mice treated with saline and unstressed controls (Ctrl) are shown in **(A)**; mGlu2 protein levels in the hippocampus of CRS mice treated i.p. with saline, MF (3 mg/kg), LAC (30 mg/kg) or their combination are shown in **(B)**, where the immunoblot is representative of three blots used for the analysis and contains two samples from Ctrl mice for comparative purposes; NFκB p65 protein levels in the hippocampus of Ctrl mice and CRS mice treated with saline or MF (3 mg/kg) are shown in **(C)**. Data are means ± SEM **(A)**, Student’s t test, *p < 0.05 vs. Ctrl, t_(12)_ = 2.302 (*n* = 7) **(B) (C)** One-way ANOVA + Fisher’s LSD **(B)** *p < 0.05 vs. saline and LAC, F_(3,25)_ = 2.904; *p* = 0.0546 (*n* = 4–8) **(C)** *p < 0.05 vs. all other values, F_(2,11)_ = 31.74; *p* < 0.0001 (*n* = 4–5).

Using hippocampal protein extracts, we extended the analysis to the mGlu2 receptor, which is an established epigenetic target of LAC. Immunoblot analysis showed a band at 100 kDa corresponding to the monomeric form of mGlu2 receptors, and a higher molecular weight band (approximately, 200 kDa), corresponding to receptor dimers (see Figure 4A). Exposure to stress caused a significant reduction of mGlu2 receptor protein levels in the hippocampus (Figure 4A). Treatment of CRS mice with the combination of LAC with MF significantly enhanced mGlu2 protein levels, whereas no effects were seen with either LAC or MF alone (Figure 4B). To unravel a potential mechanism underlying the synergism between LAC and MF, we moved from the evidence that LAC enhances the acetylation of p65/NFκB in different brain regions, including the hippocampus (Chiechio et al., 2006; Cuccurazzu et al., 2013; Nasca et al., 2013). Interestingly, treatment with MF (3 mg/kg) in CRS mice caused a large increase in p65/NFκB protein levels (Figure 4C), indicating that MF enhances the availability of one of the substrates for LAC-induced acetylation.

### Synergism Between L-acetylcarnitine and L-methylfolate on Structural Plasticity of Human iPSC Derived Dopaminergic Neurons

**FIGURE 5:**
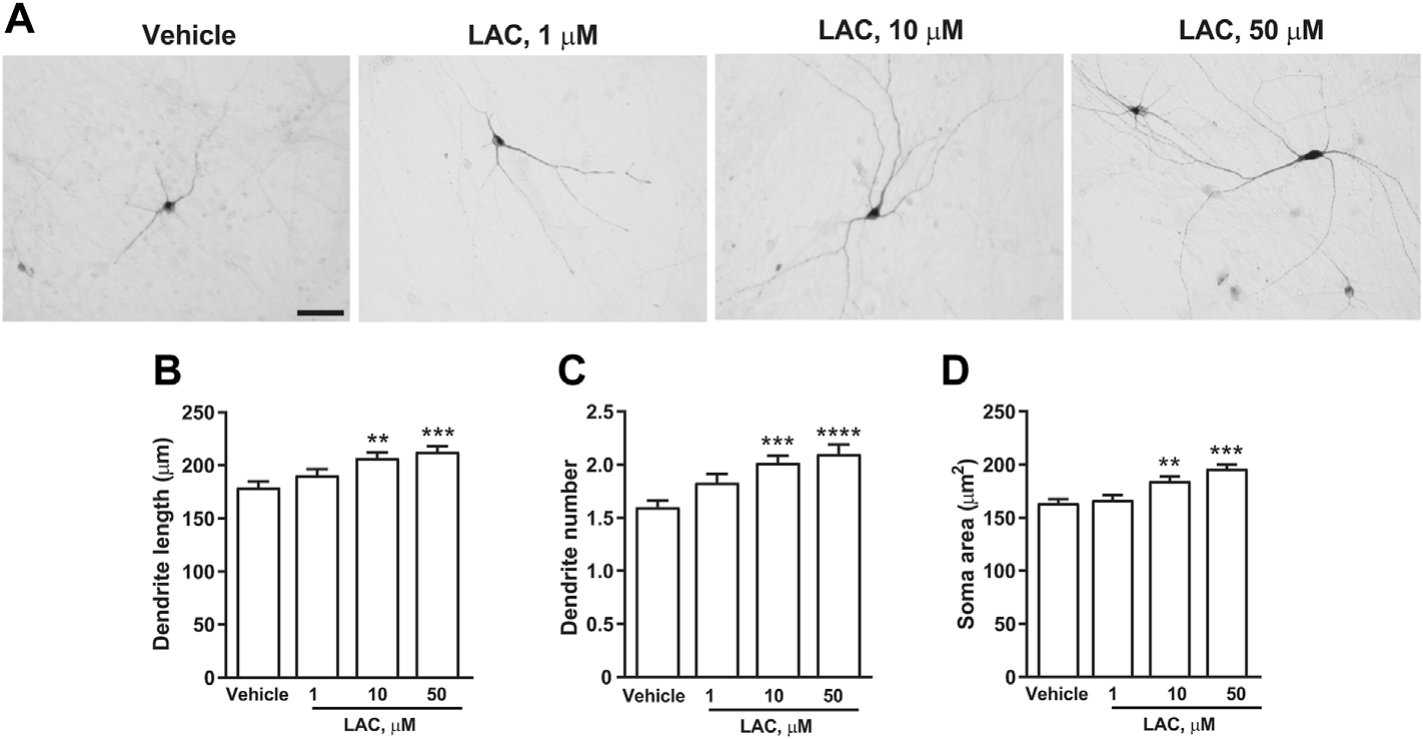
LAC promotes structural plasticity of human iPSC-derived DA neurons. Representative photomicrographs of human F3 DA neurons (40 days in culture) 72 h after exposure to vehicle or LAC (1, 10, 50 μm) are shown in **(A)**. Scale bar: 50 μm. Treatment effect on maximal dendrite length, number of primary dendrites and soma area of human F3 DA neurons is shown in **(B–D)**. Values are means ± S.E.M. (****p < 0.0001; ***p < 0.001; **p < 0.01 vs. vehicle; One-way ANOVA + Fisher’s LSD) **(B)** F_(3,396)_ = 7.674; *p* < 0.0001 **(C)** F_(3,396)_ = 8.643; *p* < 0.0001 **(D)** F_(3,396)_ = 14.44; *p* < 0.0001.

**FIGURE 6:**
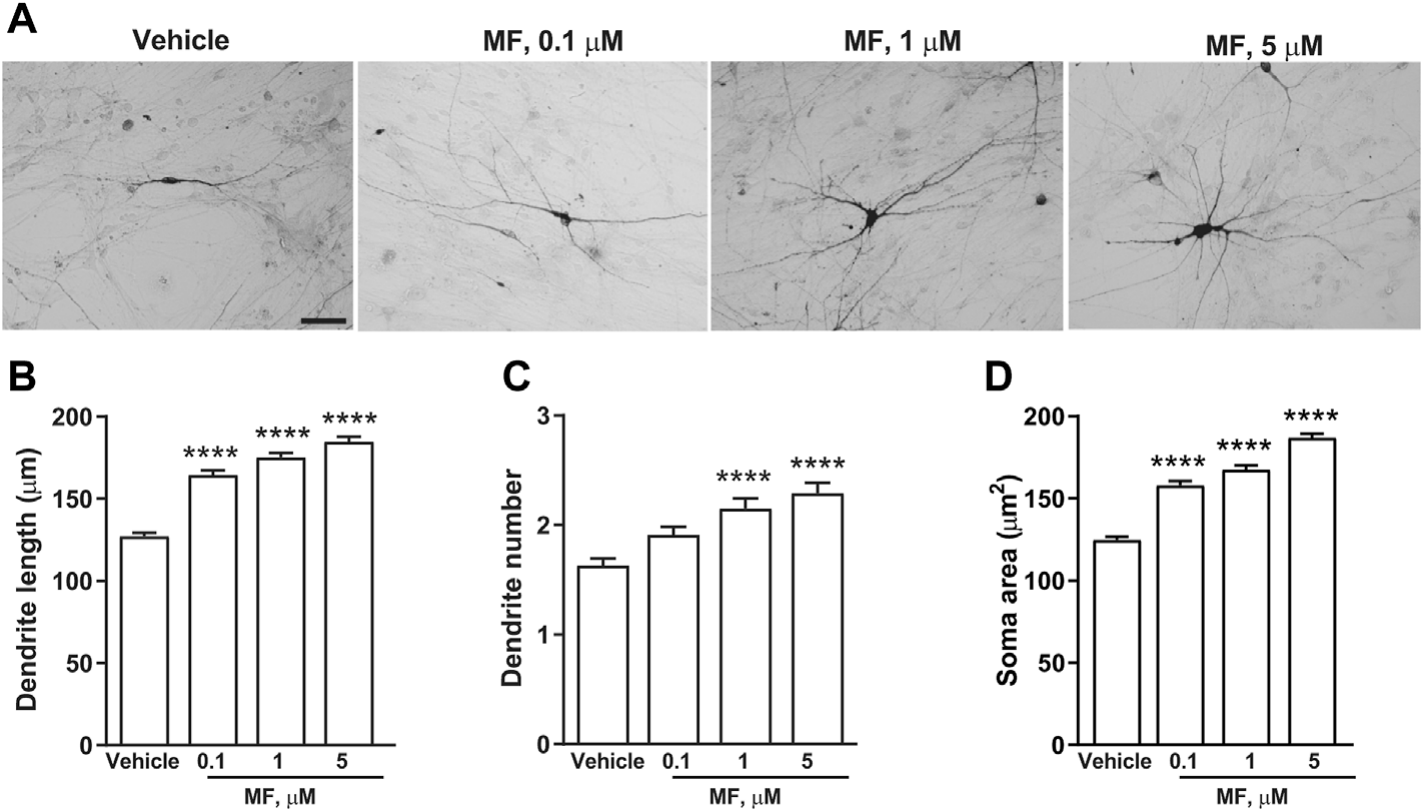
MF promotes structural plasticity of human iPSC-derived DA neurons. Representative photomicrographs of human F3 DA neurons (40 days in culture) 72 h after exposure to vehicle, or MF (0.1, 1, 5 μm) are shown in **(A)**. Scale bar: 50 μm. Treatment effect on maximal dendrite length, number of primary dendrites and soma area of human F3 DA neurons are shown in **(B–D)**. Values are means ± S.E.M. (****p < 0.0001; ***p < 0.001; **p < 0.01 vs. vehicle; One-way ANOVA + Fisher’s LSD) **(B)** F_(3,396)_ = 33.99; *p* < 0.0001 **(C)** F_(3,396)_ = 12.61; *p* < 0.0001 **(D)** F_(3,396)_ = 9.332; *p* < 0.0001.

In a first set of experiments DA neurons derived from hiPSCs (Collo et al., 2018) were exposed to LAC (1, 10, or 50 μm) for 72 h, while parallel samples were exposed to vehicle. Dopaminergic neurons were visualized using an anti-TH antibody (Figure 5A). LAC exposure produced a concentration-dependent effect on structural plasticity 72 h after the beginning of treatment increasing the maximal length of dendrites, the number of primary dendrites, and the soma area. Fishers LSD test indicated significant increases with the concentrations of 10 and 50 μm (Figures 5B–D). In a second set of experiments, DA neurons were exposed to MF (0.1, 1, or 5 μm) or vehicle for 72 h (Figure 6A) MF exposure produced a concentration-dependent effect on structural plasticity 72 h after the beginning of treatment increasing the maximal length of dendrites, the number of primary dendrites, and the soma area. Fishers LSD test indicated significant increases with all the concentrations (Figures 6B–D).

**FIGURE 7:**
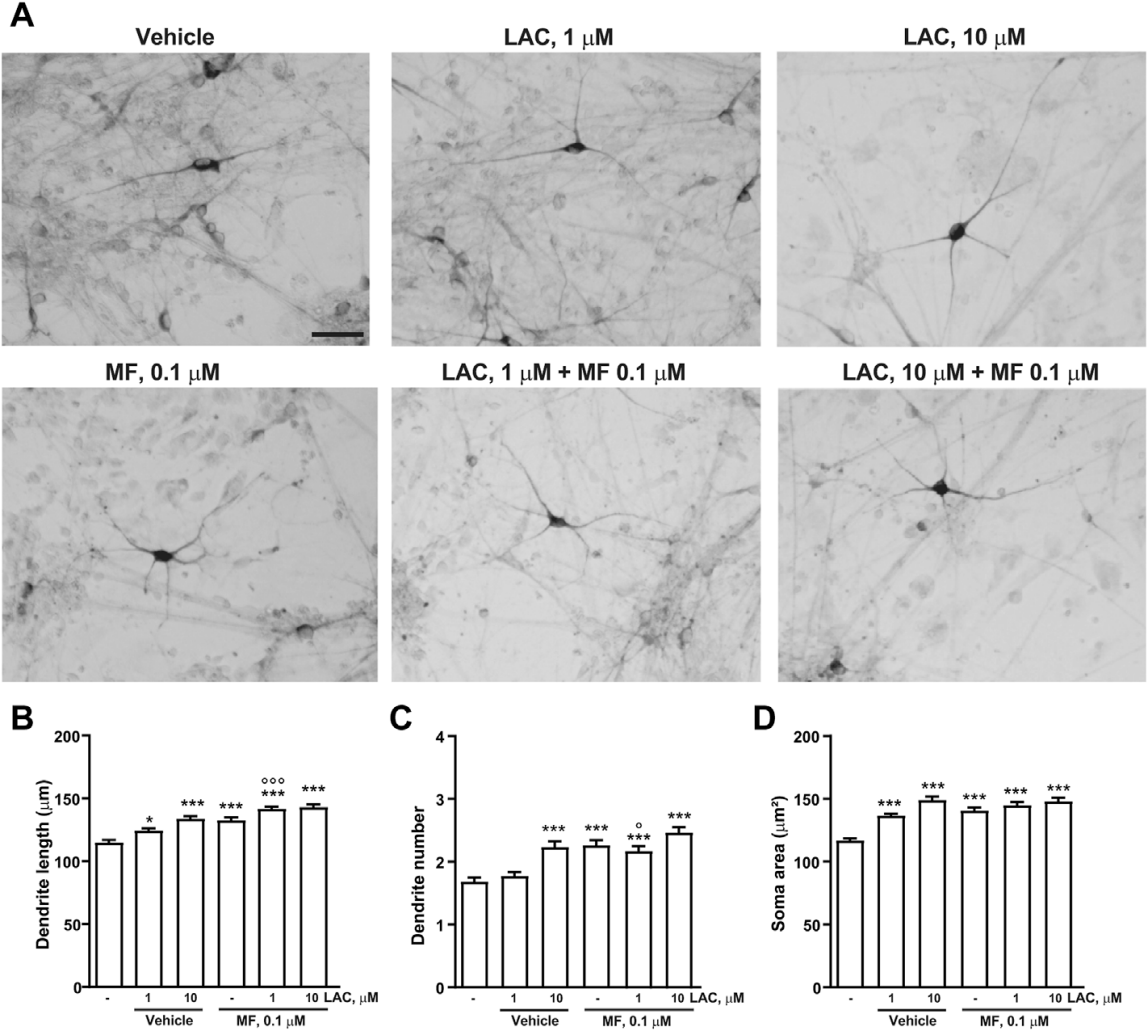
Combination treatment with LAC and MF on human iPSC-derived DA neurons. Representative photomicrographs of human F3 DA neurons (40 days in culture) 72 h after exposure to vehicle, LAC (1, 10, 50 μm), MF (0.1, 1, 5 μm) and the combination of LAC and MF are shown in **(A)**. Scale bar: 50 μm. Treatment effect on maximal dendrite length, number of primary dendrites and soma area of human F3 DA neurons is shown in **(B–D)**. In all panels, values are represented as mean ± S.E.M. (***p < 0.001; **p < 0.01; *p < 0.05 vs. vehicle; ^∘∘∘^ *p* < 0.001; *p* < 0.05 vs. LAC 1 μm; One-way ANOVA + Fisher’s LSD) **(B)** F_*(5,594)*_ = 21.75; *p* < 0.0001 **(C)** F_*(5,594)*_ = 13.67; *p* < 0.0001 **(D)** F_*(5,592)*_ = 22.31; *p* < 0.0001.

The combined effects of LAC (1 or 10 μm) and MF (0.1 μm) on dendrite growth and soma area were measured 3 days after exposure to the two compounds. A synergism between LAC and MF on dendritic length was disclosed by combining the inactive concentration of LAC (1 μm) with the lowest concentration of MF (0.1 μm). This combination increased dendritic length to a greater extent as compared to LAC or MF alone, reaching the same effect as 10 μm LAC (Figures 7A–D).

## Discussion

Using the mild CUS model of stress-related disorders in mice, we could confirm the antidepressant-like activity of LAC in the forced swim test after 9 days of treatment at the dose of 100 mg/kg, i.p. (Cuccurazzu et al., 2013; Nasca et al., 2013; Bigio et al., 2016; Nasca et al., 2017). However, this dose of LAC was ineffective after 3 days of treatment, as opposed to data obtained using FSL rats as a model of spontaneous depression (Nasca et al., 2013). The greater sensitivity of FSL rats to LAC treatment might reflect the low endogenous levels of LAC found in the hippocampus and prefrontal cortex of this strain of rats (Nasca et al., 2013). However, after 3 days of treatment we could demonstrate a strong synergism between LAC and two doses of MF in reducing the immobility time in mice exposed to CUS. The synergism was prominent with 3 mg/kg of MF. This dose roughly corresponds to the dose of MF currently used in humans (Nair and Jacob, 2016), and, therefore, this finding has potential translational value for the treatment of stress-related disorders, including MDD. We extended the analysis to CRS mice as a second model of stress. In our hands, mild CUS and CRS differed in both the behavioural and biochemical outcomes, because the immobility time in the forced swim test was increased, and BDNF levels in the frontal cortex and hippocampus were reduced, 1 week after the end of CRS, but not 1 week after the end of CUS. This might reflect the different nature of the two stress procedures (Qiao et al., 2016), and the design of our experiments, which did not differentiate mice that were vulnerable and resilient to stress. In spite of these limitations, we could demonstrate the synergism between LAC and MF in both stress models after 14 days of treatment, using a dose of LAC (30 mg/kg, i.p.) that was inactive on its own. In both stress paradigms, the sub-threshold dose of LAC enhanced BDNF and mGlu2 receptor expression in the frontal cortex and hippocampus only in combination with MF, except in the hippocampus of mice exposed to CUS, where LAC alone was sufficient to enhance BDNF levels. BDNF is a key player in mechanisms of synaptic plasticity underlying the therapeutic efficacy of antidepressants (reviewed by Björkholm and Monteggia, 2016; Yang et al., 2020), and plasma BDNF levels are enhanced in patients affected by MDD responding to medication (Zhou et al., 2017). A recent article demonstrates that most antidepressants, including SSRIs, SNRIs, and ketamine, enhance the activity of the BDNF receptor, TrkB, and mutations of the TrkB antidepressant-binding site impairs responses to antidepressants (Casarotto et al., 2021). mGlu2 receptors are metabotropic glutamate receptors coupled to Gi proteins, which are presynaptically localized and negatively modulate neurotransmitter release (reviewed by Nicoletti et al., 2011). Expression levels of mGlu2 receptors in the hippocampus and prefrontal cortex have been associated to mechanisms of resilience to stress in mice, and mGlu2 receptors are considered as potential markers of individual susceptibility to stress-related disorders (Nasca et al., 2015). Thus, our biochemical data fully support the antidepressant activity of the combination between LAC and MF. The molecular mechanism(s) underlying the synergism between LAC and MF in mice exposed to stress remain(s) to be elucidate. However, it was interesting to find that MF was able to dramatically up-regulate the expression of p65/NFκB in the hippocampus. p65/NFκB is an established substrate for LAC-induced acetylation, and levels of acetylated-p65/NFκB were found to be increased by LAC treatment in the hippocampus and prefrontal cortex of FSL rats (Nasca et al., 2013). Thus, MF might potentiate the action of LAC by enhancing the availability of one of the major substrates for LAC-induced acetylation. This hypothesis warrants further investigation.

DA has a key role in the pathophysiology of MDD, and anhedonia, a core symptom of MDD, is associated with abnormalities in mesolimbic dopaminergic transmission (Nestler and Carlezon, 2006). Drugs that activate dopaminergic transmission can promote structural plasticity in cultured DA neurons through the activation of D3 receptors, with ensuing stimulation of Akt/mTORC1 signalling (Collo et al., 2013). Interestingly, ketamine was also able to enhance structural plasticity in mouse mesencephalic and hiPSC-derived DA neurons (Cavalleri et al., 2018), suggesting that cultured DA neurons represent a model with pharmacological validity for the screening of antidepressant medications. Using hiPSC-derived dopaminergic neurons we could demonstrate a synergism between LAC and MF in stimulating dendritic growth and increasing the size of the cell soma, two effects that can also be induced by ketamine and dopaminergic agents (Collo et al., 2013; Cavalleri et al., 2018).

Taken together, our findings suggest that the combined use of LAC and MF may be valuable in the treatment of MDD, perhaps as an adjunctive therapy with conventional antidepressants, such as SSRIs or SNRIs. LAC and MF show an excellent profile of safety and tolerability, and have an optimal PK profile, with no interference with drug metabolism and efflux pumps. The “epigenetic” mechanism of LAC and MF suggests that the two drugs act at the core of the pathophysiology of MDD and perhaps other stress-related disorders, inducing rapid and long-lasting therapeutic effects. This novel form of intervention is predicted to be effective in the management of patients with TRD, in which non-monoaminergic drugs have better chances of success. Our findings lay the groundwork for an in-depth analysis of the synergism between LAC and MF in additional preclinical models, such as 1) models that are able to identify groups of animals resilient to both acute (Torrisi et al., 2020) and chronic (Dziedzicka-Wasylewska et al., 2021) stress; 2) models in which LAC and MF are combined with subthreshold doses of conventional antidepressants (e.g., SSRIs or SNRIs); and, 3) experimental animal models of TRD, characterized by a reduced or absent response to antidepressants (Planchez et al., 2019; Kelly et al., 2022).

## Data Availability

The raw data supporting the conclusions of this article will be made available by the authors, without undue reservation.
